# Prevalence, psychosocial correlates of youths’ suicidal behaviors and perspectives on the phenomena at Zagazig University: a mixed-methods study

**DOI:** 10.1186/s43045-022-00250-9

**Published:** 2022-11-02

**Authors:** Mai Mohammed ElSayed Abozaid, Mona Mostafa Aboserea, Safaa Mohammed Metwally, Hanem Ahmed AbElkhalek

**Affiliations:** 1 Psychiatric and Mental Health Nursing, Faculty of Nursing, Zagazig University, Zagazig, Egypt; 2 Public Health and Preventive Medicine, Faculty of Medicine, Zagazig University, Zagazig, Egypt

**Keywords:** Youths’ suicidal behaviors, Quality of life, Social problem-solving, Mixed-methods study

## Abstract

**Background:**

Suicidal behaviors are major public health concerns that affect large numbers of youth, leaving not only the youth but also their parents, family, friends, and peers in constant wailing. This study aimed to investigate the prevalence, psychosocial correlates, and perspectives of youths’ suicidal behaviors. A concurrent mixed-methods descriptive study was used in carrying out this study. This study was conducted at Zagazig University, Al Sharkia Governorate. A stratified multi-stage cluster sampling technique was used to enroll 364 youths. Four tools were used to collect quantitative data. They were as follows: The Youth Profile Questionnaire, composed of two parts (socio-demographic data and youth characteristics), the Suicide Behaviors Questionnaire-Revised (SBQ-R), Social Problem-Solving Inventory-Revised Short Form (SPSI-R-SF), and the Short Version of the World Health Organization Quality of Life (WHOQOL-BREF). For the qualitative part, focus group discussions were conducted to explore youths’ perspectives on the phenomenon of suicidal behaviors.

**Results:**

Quantitative findings revealed that 25% of the youth participants had a significant risk for suicidal behaviors. Being female, in the first years of practical faculties and having no friends are significantly correlated with suicidal behaviors. Further, the quality of life had a statistically significant negative correlation with suicidal behaviors. Qualitative findings were discussed under one main category: youths’ perspectives about suicidal behaviors (pressure/escaping tool, seeking help/ending pain, attention-grabbing behavior, and stain for life).

**Conclusions:**

According to this mixed-methods study, youths’ suicidal behaviors are prevalent multifaceted phenomena that certain factors have been correlated with. It is suggested that female sex, having no friends, and academic stressors are risk factors for suicidal behaviors. Also, the quality of life was introduced as a protective factor against suicidal behaviors. Therefore, it is recommended to implement prevention and management approaches to realize the complexity of the phenomena of suicidal behaviors among youth; these approaches target the youths themselves (mental health promotion and strategies for coping with stress) and the population (careful media coverage, limit access to suicidal methods, and raise the awareness about mental illness).

## Background

Suicide remains to be a problematic issue that has been considered a taboo topic all over the world especially in conservative countries. It is the 15th leading cause of death globally that accounts for 1.4% of all deaths worldwide, and also, it is estimated that about 1.5 million people will die due to suicide by the year 2020 [1]. It is prevalent among all age groups in the population, yet youths by nature are more attracted to suicide and its related behaviors mainly due to the unstructured nature of this age period and the many changes taking place in several life domains of the youths as well as a feeling “in between” adolescence and adulthood, which is an ambivalence toward adult status [2].

Suicidal behaviors, which encompass a range of phenomena such as suicidal thoughts or ideation, plans, and attempts, are pictured as significant predictors of completed suicide among youths which are estimated to be the second leading cause of death in the same age group leaving not only the youth but also their parents, family, friends, and peers in constant wailing [3]. A multinational study carried out on university students in 12 Muslim-Majority Countries, including Egypt, revealed that among 635 Egyptian youths (328 females and 324 males), about 17.5% of them had suicidal ideations and about 7.1 had attempted suicide [4].

In all settings, the suicidal phenomenon is described as a multi-determined process, that is to say, numerous factors, namely, biological, psychological, cultural, historical, and societal correlate with a person’s decision to think, plan, or attempt suicide [3]. Identifying and understanding suicidal behaviors’ correlates can be an important tool for the planning of prevention and protection activities in both clinical samples and potentially more broadly in young people, maximizing the chances of achieving the goal of zero suicide deaths in a population at markedly increased risk [5].

Socio-demographic factors are significant correlates that have promoted many theories in the field of suicidality like Shneidman’s psychache theory and Joiner’s Interpersonal Theory of suicide to adopt it as a significant correlate to suicidal behaviors as these factors have the ability to completely depict events [6]. Common demographic correlates to suicidal behaviors are (1) female gender that is positively associated with suicidal behaviors particularly suicidal attempts as females have a higher rate of suicidal attempts than males, (2) adolescence and young adulthood, and (3) college and university students [7].

Problem-solving skills defined as “the ability to describe a problem, create viable solutions, and pick a solution for implementation” have been positively associated with beneficial health-related behaviors (e.g., smoking cessation) [8]. The same is found in mental health; this skill has been shown to serve as a significant protector against numerous psychological problems, such as hopelessness, depression, and suicidal behaviors. Thus, persons with higher suicidality risk will exhibit impaired problem-solving ability and increased irritability, sadness, passivity, and avoidance in their approach to problem-solving, while those who are resilient against suicidality are more flexible when facing problems, are courageous to confront with, and are able to regulate their own emotions, neutralize negative thoughts, and trust in their abilities to cope with [9].

Recently, QOL catch the interest of the global research movement in various contexts, of which are suicidal behaviors [10]. Youth QOL is a key indicator of mental health and is positively related to a broad spectrum of positive personal, psychological, behavioral, social, interpersonal, and intrapersonal outcomes. Besides, it is widely recognized as an independent predictor of morbidity and mortality indices, including suicidality. QOL has a significant negative relationship with suicidal behaviors where poorer QOL is associated with higher odds of suicide ideation onset and is more likely to endorse suicidal attempts [11].

Taking into consideration that Egypt is the first Arab country in the number of suicide with 3799 suicides in 2016, most of them is among the youth age groups [12]. Also, the immense toll of suicide on any given society such as the decrease in population longevity, and the direct economic costs which are small in comparison to the intangible costs like grief and bereavement of family and friends [13], the authors considered the present study to highlight this important problem.

### Aim of the study

To investigate the prevalence, psychosocial correlates, and perspectives of youths’ suicidal behaviors

This aim has been achieved through the following objectives:

- Assess the prevalence of suicidal behaviors including suicidal ideation and attempts among youths.
- Identify the psychosocial factors associated with suicidal behaviors among youth.
- Explore youths’ views and perspectives of suicidal behaviors.

## Methods

### Research questions

- What is the prevalence of suicidal behaviors among youths?
- What are the psychosocial factors associated with suicidal behaviors among youth?
- How do youths perceive the phenomena of suicidal behaviors?

### Research design

**Fig. 1:**
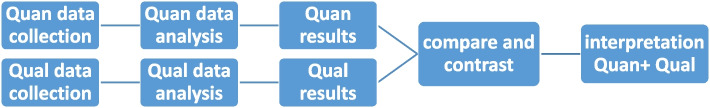
Framework of mixed-methods research design used in the current study

A concurrent mixed-methods design (Fig. 1) was adopted for this study for a more clear and comprehensive substantive understanding of youths’ suicidal behaviors [14].

### Subjects

A total of 364 youths from the Zagazig University campus were enrolled using a stratified multi-stage cluster sampling technique, and the mean age was 20.8 (SD=1.9). The faculties were stratified by type (theoretical and practical) and also by grade (all grades and post-graduate youths were included). For the selection of the faculties (stage 1), two faculties from each stratum were randomly selected, and for the selection of youths (stage 2), youths were selected from each of the strata randomly. To fulfill the required sample size (364), each of the selected strata would provide an average of 91 youths. The number of youths from each faculty has been taken with fixed allocation other than proportionate for better statistical reliability.

### Sample size

Assuming that the estimated prevalence of suicidal behaviors among youth was 22.1% (Eskin et al., 2019) [4], the sample size was calculated to be 364 youths. The sample size was calculated by using the Open-Epi software package, a confidence level of 95%, and 5% absolute precision, with a design effect of 1.5 for multistage cluster sampling. The sample size was 330; this was increased to 364 to compensate for a non-response rate of about 10%.

Tools of the data collection

### Quantitative part

Four tools were used for data collection.

#### Tool I: Youth profile questionnaire

It was developed by the researchers in light of the current related literature and composed of two parts: socio-demographic data and youth characteristics.

Part 1: Socio-demographic data: It involved two parts:

- Youth’s data: such as age, sex, residence, marital status, and occupation.
- Family’s data: such as parents’ educational level, occupation, parents’ marital status, and socio-economic level of the family.

Part 2: youth characteristics:

It involved regular physical activity, hobbies, interests, and regular number of friends.

#### Tool II: The suicidal behaviors questionnaire-revised (SBQ-R)

This questionnaire was developed by Osman et al. [15] to assess suicidal behaviors including ideations and attempts. It consists of 4 subscales, each tapping a different dimension of suicidal behaviors. The first subscale taps into lifetime suicide ideations and/or attempts rated. The second subscale assesses the frequency of suicidal ideation over the past 12 months. The third subscale taps into the threat of suicide attempts rated. The fourth subscale evaluates the self-reported likelihood of suicidal behavior in the future.

##### Scoring system

The SBQ-R consists of 4 subscales. The first subscale is rated on a 4-point Likert scale ranging from (1) never to (4) I've tried to kill myself. The second subscale is rated on a 5-point Likert scale ranging from (1) never to (5) many times. The third subscale is rated on a 3-point Likert scale ranging from (1) no to (3) yes, more than a time. The fourth subscale is rated on a 6-point Likert scale ranging from (0) never to (6) most likely. All the scores circled/checked by the respondents were summed up. The total score should range from 3 to 18. A high score (≥7) means increased suicidal behaviors, and a low score (<7) means decreased or no suicidal behaviors. Its Cronbach’s *α* was 0.76*.*

#### Tool III: Social problem-solving inventory-revised short form (SPSI-R-SF)

It was developed by D’Zurilla et al. [16] to assess cognitive, behavioral, and emotional responses to real-life problems and challenges. It consists of 25 items divided on five subscales with each subscore containing five items: positive problem orientation (PPO) (2, 4, 6, 13, 25), negative problem orientation (NPO) (1, 3, 8, 12,22), rational problem-solving style (RPS) (7, 16, 20, 21, 24), impulsivity/carelessness style (ICS) (5, 10, 14, 19, 23), and avoidance style (AS) (11, 15, 17, 18, 9). The English version was used after it was translated into Arabic by the authors, as it was not possible to reach an approved Arabic version.

##### Scoring system

The SPSI-R-SF was scored on a 5-point Likert scale, ranging from (0) not at all true to (4) extremely true. Each subscale score ranging from 0 to 20, higher scores on PPO and RPS, and lower scores on NPO, ICS, and AS indicate good social problem-solving. The scores for 15 negatively worded items (NPO, ICS, and AS) were reversed for all analyses to allow higher total scores to represent higher levels of social problem-solving. The total scores range from 0 to 100, with a cutoff point of 50%. The total score was converted into a percent score. Social problem-solving is considered to be good if the percentage is 75% or more, average if from 50 to 75%, and poor if less than 50%. Its Cronbach’s *α* was 0.76*.*

#### Tool IV: The short version of the World Health Organization quality of life (WHOQOL-BREF)

It was developed by WHO [17] to assess the quality of life of the participants and comprises 26 items, with 24 of these items grouped into four domains as follows: physical health (3, 4, 10, 15, 16, 17, 18), psychological health (5, 6, 7, 11, 19, 26), social relationships (20, 21, 22), and environmental health (8, 9, 12, 13, 14, 23, 24, 25), with two individual items assessing the perception of overall QOL and general health. The English version was used after it was translated into Arabic by the authors as the available Arabic version on the Internet was not created by the issuing authority which is WHO.

##### Scoring system

The 26 items are rated on a 5-point Likert scale, ranging from (1) very dissatisfied to (5) very satisfied. The total scores range between 26 and 130, with higher scores indicating a higher level of QOL, with a cutoff point of 50%. A total percent score of 75% or higher was considered as good, average if from 50 to 75% while a score < 50% was considered as poor. Its Cronbach’s *α* was 0.85*.*

#### Qualitative part

##### Focus group discussions (FGDs)

Research team members developed the focus group discussions. It included open-ended questions as “a close friend told you that he is really thinking of ending his life. What is your perception about him/her?”, “how do you view the phenomena of suicidal behaviors?”, and “suppose that you are in charge, what are you going to do to deal with this phenomenon?”. Each FGD consisted of 12 participants who lasted between 60 and 90 min. They were audio-recorded and transcribed verbatim. The items also dealt with the methods of suicide, gender differences, and media representation of the phenomena.

### Pilot study

Before beginning the actual study, the researchers conducted a pilot study on 10% of the youths. It was done to assess the study questionnaire’s clarity, ease of use, and feasibility, as well as to estimate the time required to complete it as well as to discover the best way to initiate and facilitate the FGD. There were no changes made to the questionnaire or the FGDS. The youths who participated in the pilot study were included in the study’s main sample.

### Content validity and reliability

The content validity of the tools utilized in this study was established by three experts in psychiatric and mental health nursing and community health nursing. They assessed the tools for applicability, clearness, comprehensiveness, understanding, relevance, and easiness for implementation. The researchers translated the study tools into the Arabic language using the translation-back translation technique to confirm their original validity. The reliability of the utilized tools was estimated by Cronbach’s alpha test in the IBM SPSS Statistics for Windows Version 27. They showed a good level of reliability.

### Trustworthiness

The researchers used four criteria of Lincoln and Guba [18] to establish the trustworthiness of the qualitative part of the study. The credibility was established through observation of non-verbal communication and member checking, while the transferability was achieved through the dense description. Field notes were made throughout the study to achieve dependability, and the advisory team provided their expertise as auditors. Confirmability of the analysis was established by using an analysis audit trail, and the findings were supported by a literature control.

### Fieldwork

the current mixed-methods study approach was designed as a “concurrent QUAl + QUANT” study, that is, quantitative and qualitative data were collected at the same time. An introductory letter including clearance for ethics was presented to the deans of the selected faculties. After permission was granted, youths within these faculties were approached. Youths who gave written consent were scheduled for focus group discussions, and after completing the discussions, they were given the quantitative questionnaire. Discussions were conducted in locations of the participants’ convenience such as lecture halls and cafeterias. All focus group discussions and quantitative data collection were conducted at the end of lectures for the day. The focus group discussion and the questionnaire took approximately 2 h (60–90 min for discussion, 30–45 min for the questionnaire). Each focus group contained 8–12 participants, as far as possible from the same friendship groupings to encourage openness and honesty. The fieldwork of the current study extended throughout the academic year 2020\2021. All focus group discussions were held in the native language and were translated to English and back-translated by the first author. All participants who were approached agreed to join the study.

### Data analysis

*Quantitative* data entry and analysis were done using IBM SPSS Statistics for Windows Version 27. Data were presented using descriptive statistics in the form of frequencies and percentages for qualitative variables, and means and standard deviations and medians for quantitative variables. Cronbach’s alpha coefficient was calculated to assess the reliability of the scales through their internal consistency. The comparisons of continuous and categorical variables by using chi-square and *T* test. In order to identify the independent predictors of various parameters’ scores, multiple linear regression analysis was used and an analysis of variance for the full regression models was done. Statistical significance was considered at *P* value <0.05.

*Qualitative* data commenced by transcribing the focus group discussions and then was analyzed using thematic analysis. The first author was in charge of the analysis. First and foremost, as authors, reading the transcripts independently, this was done while listening to the audiotapes for omissions and misprints, as well as going over the field notes for each conversation. Initial ideas and thoughts were documented at this stage of analysis. All authors individually formed these first notions into codes and discussed them. The transcripts were searched for agreed-upon codes that were related to the goal of the current study, and similar ones were structured into themes to better explain open areas of the data. The next step was to look at the links between the many themes that had emerged. All authors discussed themes that were well-established and defined. Finally, to aid the study, exemplary quotes that represented features of the concepts were chosen [19].

## Results

### Quantitative results

As to the demographic data and characteristics of the participating youths, 69.2% of participating youths were females and the mean age was 20.8±1.9. Forty-eight percent of them were in the first and second grades of college, 67% of them were single, and 81% were not working. Considering the physical activity and hobbies, 58.8% and 55.5% reported no regular physical activity and no specific hobbies, respectively. However, 60.4% reported having different interests. For the number of friends, 47.5% had four or more.

The prevalence rates of suicidal behaviors are presented in Table 1. Overall, 25.0% of the studied youths scored above the cutoff point of ≥7 on the SBQ-R, indicating a significant risk for suicidal behavior. Examination of individual items for the whole sample revealed that 29.9% of youths considered the idea of suicide with 11.8% who already attempted suicide. Moreover, 39.3% of the participating youths thought about ending their lives during the past year. Finally, 8.7% of the studied youths reported the likelihood of attempting suicide in the future.

**Table 1:** Total scores of suicidal behaviors as reported by the studied youth (*n*=364)

SBQ-R	Frequency	Percent
**Lifetime suicide ideations and/or attempts**		
None-suicide group	171	47
Suicide-risk group	109	29.9
Suicide-plan group	41	11.3
Suicide-attempt group	43	11.8
**Frequency of suicidal ideation over the past 12 months**		
Never	221	60.7
Rarely (1 time)	73	20.1
Sometimes (2 times)	34	9.3
Often (3–4 times)	24	6.6
Very often (5 or more times)	12	3.3
**Threat of suicide attempt**		
None	256	70.3
Once	77	21.2
More than once	31	8.5
**Self-reported likelihood of suicidal behavior in the future**		
Never	231	63.5
No chance at all	52	14.3
Rather unlikely	39	10.7
Unlikely	10	2.7
Likely	22	6
Rather likely	6	1.6
Very likely	4	1.1
**Total score**		
Mean ± SD	5.8 ± 3.3	
Median (range)	5 (3–18)	
< 7 points	27 (75%)	
≥ 7 points	91 (25%)	

Table 2 and 3 revealed that youths with a higher risk for suicidal behaviors were statistically significantly more likely to be female (28.2%) (*P* value= 0.03), in the 1st grade (30.4) (*P* value˂ 0.04) and have no friends (47.6%) (*P* value˂ 0.001).

**Table 2:** Relation between suicidal behaviors and sociodemographic characteristics of the studied youths

Sociodemographic characteristics	Total SBQ-R				Test of sig.	*P*
	˂ 7 points		≥ 7 points			
	No	%	No.	%		
**Age (years)**					T	
Mean ± SD	20.9 ± 2.0		20.4 ± 1.7		1.9	0.06
**Sex**						
Male (*n*=112)	92	82.1	20	17.9	χ^2^	0.03*
Female (*n*=252)	181	71.8	71	28.2	4.4	
**Residence**						
Rural (*n*=201)	158	78.6	43	21.4	χ^2^	0.08
Urban (*n*=163)	115	70.6	48	29.4	3.1	
**Marital status**						
Single (*n*=244)	189	77.55	55	22.5	χ^2^	0.2
Engaged (*n*=109)	78	71.6	31	28.4	5.1	
Married/divorced (*n*=11)	6	54.5	5	45.5		
**Occupation**						
Working (*n*=70)	54	77.1	16	22.9	χ^2^	0.6
Not working (*n*=294)	219	74.5	75	25.5	0.2	
**Father education**						
Illiterate, read, & write (*n*=27)	21	77.8	6	22.2	*χ*^2^	0.3
Primary education (*n*=29)	22	75.9	7	24.1	4.1	
Secondary education (*n*=121)	83	68.6	38	31.4		
High education (*n*=187)	147	78.6	40	21.4		
**Mother education**						
Illiterate, read, & write (*n*=43)	30	69.8	13	30.2	*χ*^2^	0.8
Primary education (*n*=20)	15	75.0	5	25.0	0.9	
Secondary education (*n*=163)	125	76.7	38	23.3		
High education (*n*=138)	103	74.6	35	25.4		
**Father occupation**						
Not working (*n*=41)	32	78.0	9	22.0	*χ*^2^	0.8
Worker (*n*=21)	18	85.7	3	14.3	2.5	
Farmer (*n*=16)	13	81.3	3	18.8		
Free business (*n*=85)	63	74.1	22	25.9		
Employee (*n*=144)	104	72.2	40	27.8		
Professional (*n*=57)	43	75.4	14	24.6		
**Mother occupation**						
Working (*n*=155)	111	71.6	44	28.4	χ^2^	0.2
Not working (*n*=209)	162	77.5	47	22.5	1.7	
**Socio-economic level of family**						
Low (*n*=91)	70	76.9	21	23.1	*χ*^2^	0.4
Middle (*n*=165)	127	77.0	38	23.0	1.8	
High (*n*=108)	76	70.4	32	29.6		

*P<0.05 significant, **P<0.001 highly significant, ***P>0.05 non-significant

**Table 3:** Relation between suicidal behaviors and characteristic profile of the studied youth (*n*=364)

Youth profile	Total SBQ-R				***χ***^**2**^	***P***
	˂ 7 points		≥ 7 points			
**Grade**						
First grade (*n*=102)	71	69.6	31	30.4	4.2	0.04*
Second grade (*n*=72)	51	70.8	21	29.2		
Third grade (*n*=81)	64	79.0	17	21.0		
Fourth grade (*n*=99)	78	78.8	21	21.2		
Postgraduate (*n*=10)	9	90.0	1	10.0		
**Regular physical activity**						
Yes (*n*=150)	117	78.0	33	22.0	1.2	0.3
No (*n*=214)	156	72.9	58	27.1		
Hobby						
Yes (*n*=162)	118	78.0	44	27.2	0.7	0.4
No (*n*=202)	155	72.9	47	23.3		
**Interests**						
Yes (*n*=220)	165	75.0	55	25.0	NA	NA
No (*n*=144)	108	75.0	36	25.0		
**Number of friends**						
None (*n*=21)	11	52.4	10	47.6	10.2	˂0.001*
One (*n*=49)	32	65.3	17	34.7		
Two or three (*n*=121)	91	75.2	30	24.8		
Four or more (*n*=173)	139	80.3	34	19.7		
**Living with**						
Both parents (*n*=299)	227	75.9	72	24.1	0.8	0.7
One of the parents (*n*=54)	38	70.4	16	29.6		
Relative (*n*=11)	8	72.7	3	27.3		
**Marital status of parents**						
Married (*n*=324)	245	75.6	79	24.4	2.7	0.3
Divorced (*n*=8)	4	50.0	4	50.0		
Widow (*n*=32)	24	75.0	8	25.0		

*P<0.05 significant, **P<0.001 highly significant, ***P>0.05 non-significant

Table 4 showed that of 364 participating youths, 75.0% had high levels of social problem-solving skills and 43.1% were at a high quality of life with 86.5% reported high in the social relationship domain.

**Table 4:** Frequency distribution of the sample according to social problem-solving and quality of life

Items	Poor		Average		High	
	No.	%	No.	%	No.	%
**Social problem-solving**						
Positive problem orientation	7	1.9	43	11.8	314	86.3
Negative problem orientation	26	7.1	109	29.9	229	62.9
Rational problem-solving style	10	2.7	68	18.7	286	78.6
Impulsivity/carelessness style	47	12.9	159	43.7	158	43.4
Avoidance style	80	22.0	152	41.8	132	36.3
Total	5	1.4	86	23.6	273	75.0
**Quality of life**						
Physical health	7	1.9	181	49.7	176	48.4
Psychological health	54	12.4	198	54.4	121	33.2
Social relationships	0	0.0	49	13.5	315	86.5
Environment	38	10.4	235	64.6	91	25.0
Total	6	1.6	201	55.2	157	43.1

The correlation between study variables is declared in Table 5. Suicidal behaviors were statistically significantly negatively correlated with quality of life (*r*= −0.415) at *P*=0.001, while there was no statistically significant correlation with social problem-solving (*r*= 0.053) at *P*=0.032.

**Table 5:** Correlation matrix between study variables (*n*=364)

Variables	Suicidal behaviors		Social problem-solving		Quality of life	
	***r***	***p***	***r***	***P***	***R***	p
**Suicidal behaviors**						
**Social problem-solving**	**0.053**	**0.316**				
**Quality of life**	**−.415**	0.001**	**−.112**	.032*		

*r* correlation coefficient

*significant *P* <0.05, **highly significant *P*<0.001

**Fig. 2:**
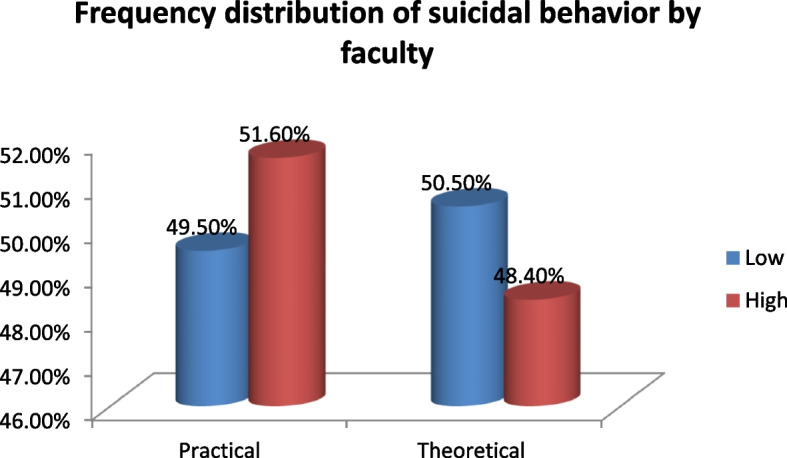
Frequency distribution of the suicidal behaviors regarding the faculty type (*n*=364)

Figure 2 reveals the distribution of the suicidal behaviors regarding faculty type (*n*=364) where youths with a higher risk for suicidal behaviors tend to study at practical faculties (51.6%) (*P* value˂ 0.02).

### Qualitative results

**Fig. 3:**
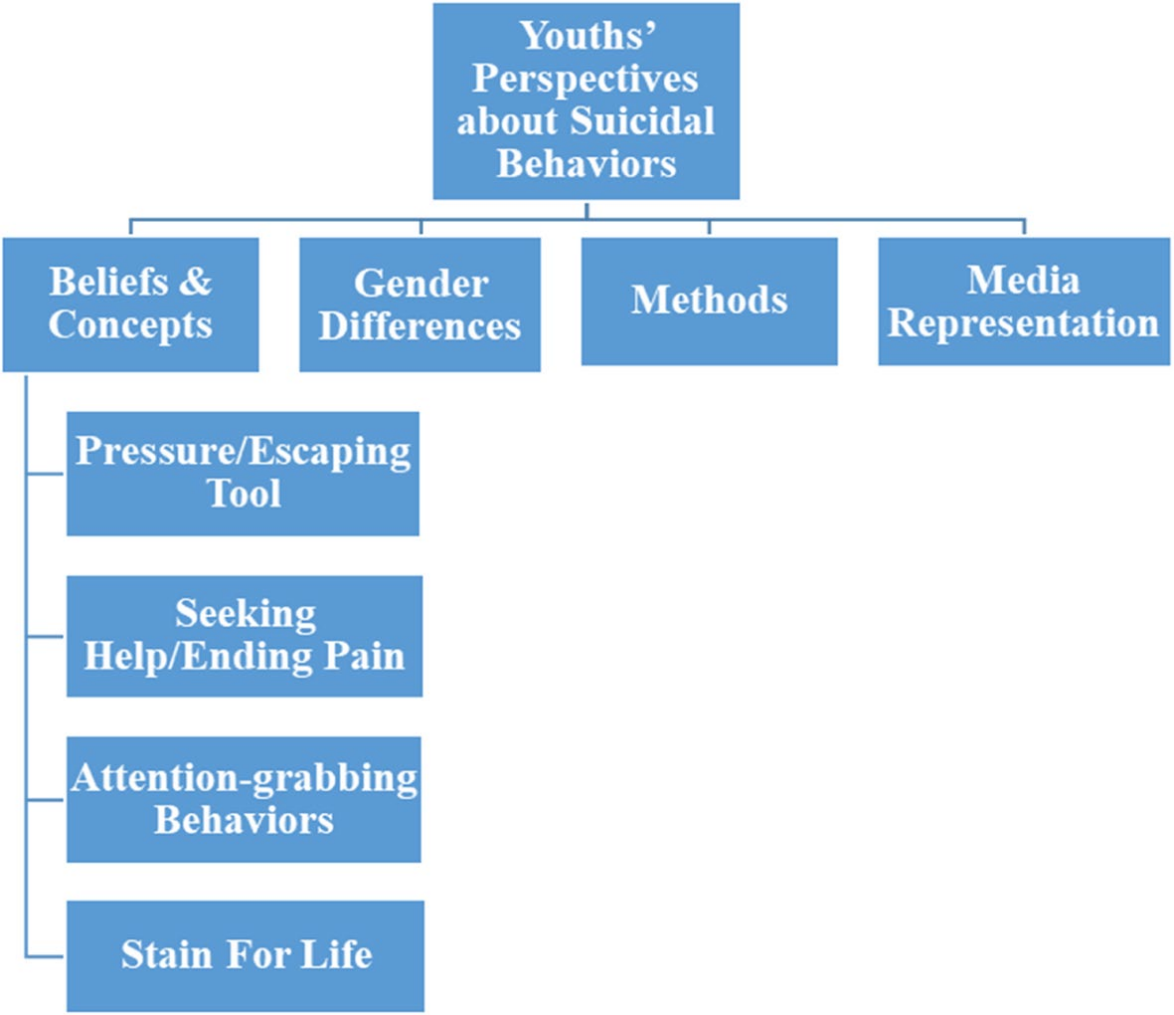
Themes and subthemes that emerged as a result of the qualitative analysis

The focus groups revealed one main theme relevant to the study’s research question “youths’ perspectives about suicidal behaviors”. Subthemes within this theme included (a) beliefs and concepts (pressure/escaping tool, seeking help/ending pain, attention-grabbing behavior, and stain for life); (b) gender differences; (c) methods used in suicidal behaviors; and (d) media representation of suicide (Fig. 3).

- *Beliefs and concepts*

Within this subtheme, youths viewed suicidal behaviors as follows:

### Pressure/escaping tool

- Quote: “My friend tried to commit suicide more than once, and each time the same method, cutting her arteries. She was trying to oblige her family to do what she wants”.
- Quote: “A girl I know, always threaten her family with suicide when she tried to avoid problems”

### Seeking help/ending pain

- Quote: “My colleague did not want to kill herself, she just wanted to end the current situation. This was the easiest way for her, there was no other way”.

### Attention-grabbing behavior

- Quote: “Disclosure of suicidal intentions means that I want an advice and I am waiting to be stopped from this act”.
- Quote: “Some persons want to be source of attention”.
- *Stain for life*
- Quote: “Whoever attempts suicide is an infidel and does not deserve our wishes to rest in peace”.

b. *Gender differences*

This subtheme discussed the differences between males and females under the points of prevalence and methods used in suicide.

- Quote: “It seems to me that no one hasn’t been in such a situation, especially females”.
- Quote: “I tried to jump in front of the train 3 times” (youth male).
- Quote: “Boys use more strong methods. I think a lot in different methods but when execution time come I get scared and use other methods like slow death as stopping eating for days or taking sedatives” (youth female).

iii. *Methods used in suicide*

Within this subtheme, the methods of suicide were extracted from suicide cases or attempts witnessed or experienced by the youths themselves.

- Quote: “Jumping in water or from bridges or heights as well as in front of trains”.
- Quote: “Swallowing pills, ingestion of pesticides by dissolving them in water particularly in our rural areas where swallowing aluminum phosphide is trending”.
- Quote: “hanging, cutting and stabbing”.

iv. *Media representation of the phenomena*

This subtheme was kind of confusing as the youths confirmed the impact of social media on the increased number of suicide cases and also could not deny its role in preventing suicide.

- Quote: “Unfortunately, social media normalize and romanticize suicide and suicidal behaviors”.
- Quote: “The media, whether it is social media, video or audio, increases suicide rates as people imitate what it shows”.
- Quote: “When the media circulates the issue of suicide, it puts pressure on those who have the idea, and it can actually push them to implement”.
- Quote: “Social media reinforces the class difference, yet it can be used to raise awareness against suicidal behavior, so it can be considered a double-edged sword”.

## Discussion

Worldwide, suicidal behaviors rank as a leading cause of death among youths aged 15–29 years, about one fifth of all deaths among youths, and are responsible for a substantial number of premature deaths as well as a huge amount of pointless suffering and societal loss of many young lives [20]. Therefore, the aim of this study was to investigate the phenomena of suicidal behaviors among youths. As what follows, both quantitative and qualitative findings of the current study are discussed in relation to findings of previous literature and other studies to reach evidence-based answers to the study research questions.

*As for the prevalence rates of suicidal behaviors*, the prevailing opinion among the participants during FGDs was that such behaviors are prevalent and are in the event of an increase. Moreover, most participants claimed that no area has not witnessed a suicidal behavior case as well as no one has not passed through suicidal behaviors’ situation. Congruently, quantitative results of the current study revealed that a quarter of the participants reported a significant risk for suicidal behaviors which is quite high and warrants immediate attention as it is an important predictor of death by suicide.

More specifically, the current study revealed that slightly higher than one tenth of youths reported previously suicide attempts, whereas nearly two fifth of the youths considered such actions within the past 12 months and that higher than one fifth told others that they would attempt suicide at least once, with one tenth having a plan of how they would do such action. However, the result of possible future attempts remains low (8.7%). These rates could be explained by assuming that the youth period naturally is full of psychological fluctuations and confusion of roles. Likewise, no one can deny the various pressures that young people go through, especially the academic and economic ones. Also, it should be noted that this study was conducted during the global epidemic of COVID-19, which might have contributed to some rise in rates.

This result was comparable to the rates estimated by Akram et al. [21] in the UK who revealed that 10.8% had previous suicide attempts, 42.2% considered an attempt through the past year, 25.1 told others about their planned attempts, and 6% may commit suicide in the future. Suicidal risk and plans were moderately high as the rates of suicidal behaviors’ risk and suicide plans were 37.3% and 20.1%, respectively.

For suicidal ideation, the current study reported that nearly one third of the studied youths had a lifetime prevalence of suicidal ideation. This was comparable to the study conducted by Eskin et al. [4] who revealed that lifetime prevalence of suicidal ideation in 12 Muslim countries including Egypt was 22.1%. However, in the study done by Hirsch et al. [22], suicidal ideation was higher than the current rate as it was 43.1%. That increase might be ought to the use of different tools, different sample sizes, and motivations of the student to respond.

*Concerning sociodemographic correlates*, the current study revealed that the highest risk for suicidal behaviors was found in youths who were more than 20 years old. These results were inconsistent with Voss et al. [23], who studied suicidal behaviors among adolescents and young adults in Germany and concluded that suicidal behaviors sharply increased at the age of 20 or less. Likewise, Thompson and Swartout [24], in their study about the epidemiology of suicide attempts among youth transitioning to adulthood, found that suicidal behaviors changed as youth matured into young adulthood, and most reduced their suicidal behavior likelihood as their risk factors correspondingly declined.

In the same vein, the current findings revealed that the risk of suicidal behaviors proportionally decreased with the progression of youths’ grades. About two thirds of the studied youths who were at high risk for suicidal behaviors were in the 1st and 2nd grades. This might be explained by the inability of the 1st and 2nd grade youths to withstand the climate of the new environment, experience a number of challenges related to accommodations, communication, transportation, social interaction, discrimination, and academic life [25].

The type of faculty in the current study was a surprising result as the rates of suicidal behaviors among youths studying at practical faculties and theoretical faculties were very close. Given the tremendous scientific pressure and long years of study in practical colleges compared to other theoretical colleges, it was expected to see very high rates of suicidal behaviors among youths studying at practical faculties. Yet, the picture became clear when youths mentioned through FGDs that a graduate of theoretical colleges holds stressors of having neither a future nor a prestigious job which is a common view of the vast majority of Egyptian families.

Moreover, this finding indicated that there was a statistically significant relationship between suicidal behaviors and sex as nearly one third of the studied female youths were at higher risk for suicidal behaviors. The discrepancy between males and females might be explained on the basis of gender role socialization theory, which states that “females are expected to be dependent and indecisive, and express their stress via rumination, so females have a higher rate of suicide attempts than males” [26]. This finding is congruent with Abdu et al. [7] who illustrated that female gender was among factors positively associated with suicidal behaviors and that the odds of females to engage in suicidal behaviors were higher compared to males and the results of Alothman & Fogarty [27] where they indicated that female are more ideators and/or attempters, yet men kill themselves more often than women. Also, the results of Abd-elaziz et al. [28] in their study of suicidal ideation among youth at Suez-canal University, Egypt, who revealed that females were twice as likely as men to think about suicide.

Further, the difference in suicidal behaviors between men and women does not stop at prevalence rates, but extends to the method each of them uses in his/her attempt to commit suicide. The participants in the study qualitatively stated that the methods used by men are the strongest, while women use less dangerous methods, likely due to the fear that possesses women. In line with this point, Eskin et al. [4] reported that more men than women use more lethal methods such as hanging, firearms, jumping, and drowning while more women than men used less lethal methods like taking pills and using a sharp instrument and that is why fewer attempts by women than men required medical attention.

In addition to the above, the number of friends comes as another important demographic indicator, as the current study showed that it was inversely proportional to suicidal behaviors. Almost half of the youths having no friends were experiencing suicidal behaviors compared to two fifth of youths with four or more friends. This can be attributed to that friends in this period of life are increasingly more important as a source of social support in the life of youths as they spend more time with their peers.

*Social proble solving* was examined as another correlation with suicidal behaviors. However, contrary to what was expected and to the IPTS, social problem-solving did not significantly associated with suicidal behaviors. This finding was not in line with Walker et al. [29] who reported that individuals with higher levels of social problem-solving ability were less likely to report suicidal behaviors and Chu et al. [30] who found significant and negative associations between social problem-solving and suicidal behaviors in 5 adult samples, providing support to the IPTS particularly to PB. This difference can be ought to both methodological and sociocultural explanations.

*For the quality of life*, the current study revealed a negative statistically significant association between suicidal behaviors and quality of life, providing support to the Interpersonal Theory of Suicidal behaviors by Joiner et al. [31] and Van Orden et al. [32] which states that poor quality of life results in a higher possibility of having suicidal behaviors and vice versa. This finding remained consistent with the studies done by Farabaugh et al. [33] and Fairweather-Schmidt et al. [34], in which they found that poorer QOL was associated with higher odds of suicide behaviors’ onset. Also, the study done by Balazs et al. [11], in which they recommended that suicide prevention strategies should involve assessing QOL particularly in cases with emotional and peer problems.

Interestingly, a substantial proportion of the participants through FGDs mentioned their negative attitudes toward suicidal behaviors. This negative attitude was easily predicted as the participant believed that suicidal behaviors could be anything but ending one’s life. Their concepts were classified into four main subthemes: pressure/escaping tool, seeking help/ending pain, attention-grabbing behavior, and stain for life. These findings go along with that of Rajappa et al. [35] in which they suggested that suicidal behaviors are attempts to escape negative emotions that occur when people lack emotion control tools when they are distressed. Also, these findings shared similarities with Singh et al. [36] results where they found that 74.6% of suicidality ideated to end pain and 12.1% to get attention. As well, these findings were in agreement with the findings of Stubbing and Gibson’s [37] study where 9 focus group discussions were conducted with youth in New Zealand. They found that suicidal behavior as an appeal for help was a valid approach by many youths and that youths seemed to view expressions of suicidality as a legitimate way of communicating distress to others.

In a similar vein, a study done by Zou et al. [38] revealed that participating youths viewed their peers’ suicidal behaviors, especially female suicidality, as a way to get attention or manipulate others and they stained their peers as weak persons and irresponsible. In addition, Moksony and Hegedus [39] reported that people tend to stigmatize suicidal persons as losing their faith, infidels, or atheists.

Qualitative study results raised the point related to media representation of suicidal behaviors particularly on social media. It was clearly evident from the FGDs that the participating youths were confused about social media roles. Phrases like “two sides of the same coin” and “double-edged weapon” were used by them. The same results were found in the study of Swedo et al. [40], where they suggested that social media are a powerful tool with the capacity to provide protective effects and meaningful interventions for those at risk of suicide as it is accessible, acceptable, and fast with which helpful messages can be transmitted along with its possible harmful effect as containing distressing or sensationalized content, normalizing suicide as a response to one’s problems, and spreading information about suicide location and methods.

## Conclusions

Taken as a whole, this concurrent triangulation reported that suicidal behaviors are a widespread, complex, and multi-faceted phenomenon that did have significant and long-lasting consequences on the youths and the community. Additionally, this approach underscored the role of psychosocial correlates in youths’ suicidal behaviors and the need of tailoring it to youths’ suicide prevention efforts. On the basis of the current study findings, the following recommendations are suggested: the need of tailoring sociodemographic correlates and quality of life in youths’ suicide prevention efforts; further research on youth suicidality to investigate more suicide correlates such as pressures and distressful feelings that they deal with; and implement prevention and management approaches realize the complexity of the phenomena of suicidal behaviors among youth and that focus on different facets other than the current emphasis on mental illness as the single most salient issue, these approaches target the youths themselves (mental health promotion and strategies for coping with stress), and population (careful media coverage, limit access to suicidal methods and raise the awareness about mental illness).

## Data Availability

As detailed in the “References” section, all data was accessible through the Internet.

## Abbreviations

SBQ-R: The Suicide Behavior Questionnaire-Revised
SPSI-R-SF: The Social Problem-Solving Inventory-Revised Short Form
PPO: Positive problem orientation
NPO: Negative problem orientation
ICS: Impulsivity/carelessness style
RPS: Rational problem-solving
AS: Avoidance style
WHOOQOL-BREF: The Short Version of the World Health Organization Quality of Life
QOL: Quality of life
FGDs: Focus group discussion
QUAL: Qualitative
QUANT: Quantitative
